# Supplementary material for: The genome sequence of the Roundleaf geranium, Geranium rotundifolium L. (Geraniales: Geraniaceae)
Source: Wellcome Open Res. 2026 Mar 18;11:168. [Version 1] doi: 10.12688/wellcomeopenres.26116.1 (PMC13096780; doi:10.12688/wellcomeopenres.26116.1)
Supplement: Peer review history

# Reviewer report: Reviewer response for version 1

**DOI:** 10.21956/wellcomeopenres.28759.r152433

**Published:** 2026-04-20

**Authors:** Margaret E Staton

**Affiliations:** 1 The University of Tennessee, Knoxville, Tennessee, USA

The authors present a chromosome scale haploid reference genome for Geranium rotundifolium. The genome used HiFi and HiC to yield a high quality final product. 

 The authors might comment on why they opted to use hifiasm with the -primary flag instead of  haplotype resolved assemblies, which are usually possible when hi-C data is included. 

 Its unclear if the gene annotation utilized the RNASeq data or if the genes were functionally annotated. There's also no information on the repetitive content. The genome was annotated by the Ensembl pipeline, but some results on these points would be a welcome addition.

Are sufficient details of methods and materials provided to allow replication by others?

Yes

Is the rationale for creating the dataset(s) clearly described?

Yes

Are the datasets clearly presented in a useable and accessible format?

Yes

Are the protocols appropriate and is the work technically sound?

Yes

Reviewer Expertise:

Computational genomics

I confirm that I have read this submission and believe that I have an appropriate level of expertise to confirm that it is of an acceptable scientific standard.

# Reviewer report: Reviewer response for version 1

**DOI:** 10.21956/wellcomeopenres.28759.r152432

**Published:** 2026-04-06

**Authors:** Nils Stein

**Affiliations:** 1 Leibniz Institute of Plant Genetics and Crop Plant Research (IPK), Seeland, Germany

The authors present a de novo nuclear and plastid genome assembly of G. rotundifolium. The species was selected as being endemic to UK and thus became a selected species within the DToL initiative. The study follows transparently the established standardized procedures and all assembly metrics and stats are presented and benchmarked against the Earth Biogenome Project standards.

 I am reviewing the first time for this format but I am absolutely thrilled - this is, to my opinion the way genome sequences should be presented and made public rapidly in order to generate impact in the science community comments:

 1) HiC interaction plot: I understand that the effort is limited that can be spent in a high throughput genome sequencing effort for individual curation steps. Individual chromosomes still have obvious traces of orientation/assembly artifacts. It also looks as if chromosomes are presented in random orientation. There is a convention that chromosomes shall be presented from short to long arm. It looks as if this species has a mix of acrocentric, telocentric and metacentric chromosomes. If there are any cytological data the chromosome order could be referred to this would add value.

Are sufficient details of methods and materials provided to allow replication by others?

Yes

Is the rationale for creating the dataset(s) clearly described?

Yes

Are the datasets clearly presented in a useable and accessible format?

Yes

Are the protocols appropriate and is the work technically sound?

Yes

Reviewer Expertise:

Plant Genetics, Plant Genomics, Plant Genetic Resources

I confirm that I have read this submission and believe that I have an appropriate level of expertise to confirm that it is of an acceptable scientific standard.
